# Metabolic Regulation in the Induction of Trained Immunity

**DOI:** 10.1007/s00281-024-01015-8

**Published:** 2024-07-25

**Authors:** Anaisa V. Ferreira, Jorge Domínguez-Andrés, Laura M. Merlo Pich, Leo A. B. Joosten, Mihai G. Netea

**Affiliations:** 1 Department of Internal Medicine and Radboud Center for Infectious Diseases (RCI), Radboud University Nijmegen Medical Center, 6500HB Nijmegen, The Netherlands; 2 Department of Medical Genetics, Iuliu Hatieganu University of Medicine and Pharmacy, Cluj-Napoca-Napoca, Romania; 3 Department for Immunology and Metabolism, Life and Medical Sciences Institute (LIMES), University of Bonn, 53115 Bonn, Germany

**Keywords:** Trained immunity, Metabolism, Epigenetics, Therapy

## Abstract

The innate immune system exhibits features of memory, termed trained immunity, which promote faster and more robust responsiveness to heterologous challenges. Innate immune memory is sustained through epigenetic modifications, affecting gene accessibility, and promoting a tailored gene transcription for an enhanced immune response. Alterations in the epigenetic landscape are intertwined with metabolic rewiring. Here, we review the metabolic pathways that underscore the induction and maintenance of trained immunity, including glycolysis, oxidative phosphorylation, the tricarboxylic acid cycle, and amino acid and lipid metabolism. The intricate interplay of these pathways is pivotal for establishing innate immune memory in distinct cellular compartments. We explore in particular the case of resident lung alveolar macrophages. We propose that leveraging the memory of the innate immune system may present therapeutic potential. Specifically, targeting the metabolic programs of innate immune cells is an emerging strategy for clinical interventions, either to boost immune responses in immunosuppressed conditions or to mitigate maladaptive activation in hyperinflammatory diseases.

## Trained Immunity as the Memory of the Innate Immune System

**Fig. 1:**
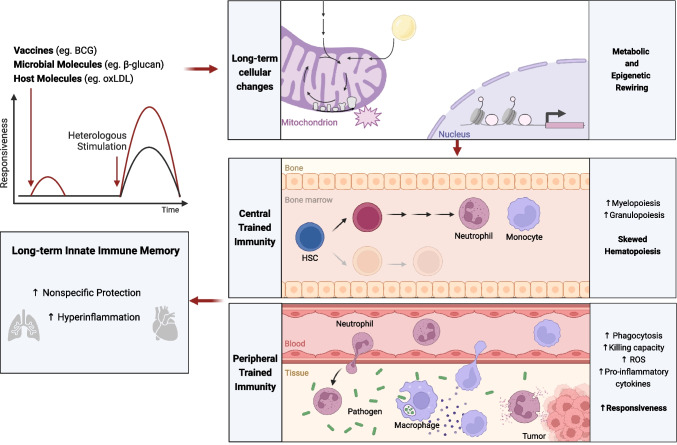
Summary of the players involved in innate immune memory. Trained immunity inducers, such as live-attenuated vaccines or microbial and host molecules may trigger metabolic and epigenetic changes. These alterations support a hematopoietic bias towards myelopoiesis and granulopoiesis (i.e. central trained immunity), which leads to the production of trained innate immune cells (eg. neutrophils and monocytes/macrophages). Trained innate immune cells show enhanced effector functions (i.e. peripheral trained immunity), which confers protection against infections or enhances antitumoral activity, or may become maladaptive in the case of hyperinflammatory conditions. Figure created with Biorender.com

The immune system consists of two components: the innate system, which provides immediate but less specific defence; and the adaptive system, which offers highly specific responses but takes days to weeks to develop. Immunological memory is classically attributed to the adaptive immune system, which allows for faster and more effective responses after repeated exposures to the same antigen. Vaccines leverage this process by inducing specific adaptive immune responses, offering long-lasting protection against targeted pathogens [1]. However, living systems without an adaptive immune system still develop increased resistance to subsequent infections. For instance, plants can establish an immune response against a pathogen that provides protection against distinct subsequent infections [2]. This systemic acquired resistance (SAR) demonstrates that the innate immune system can exhibit memory characteristics. Human and murine models also suggest that the innate immune system possesses memory features, referred to as *trained immunity* or *innate immune memory* **(**Fig. 1**)** [3]. Specifically, challenged innate immune cells acquire memory features that allow for a faster and more robust response to a secondary event in an antigen-agnostic fashion. This protection conferred by trained immunity, although of shorter duration than the adaptive immune memory, can last from months to a few years.

Different epidemiological studies have reported that live-attenuated vaccines have non-specific benefits. Namely, the widely used vaccine Bacille Calmette-Guérin (BCG), administered to target tuberculosis, has been shown to confer protection against pathogens other than *Mycobacterium tuberculosis.* Low birth-weight neonates vaccinated with BCG exhibited lower all-cause mortality, primarily through the prevention of sepsis and respiratory tract infection [4, 5]. Indeed, whole blood stimulation assays showed that BCG-vaccinated infants had increased production of cytokines upon stimulation with the fungus Candida albicans or the TLR1/2 agonist Pam3Cys [6]. In elderly individuals, BCG vaccination has also been shown to decrease respiratory infections, highlighting the potential of BCG-induced trained immunity for the broad protection of distinct vulnerable populations [7]. Live-attenuated viral vaccines, such as the oral polio vaccine (OPV) and the measles, mumps, and rubella (MMR) vaccine have also been shown to confer non-specific protection, since both OPV and MMR have reduced infant mortality, independently of the protection against the target infections [8, 9].

In addition to vaccines, other agents also induce innate immune memory programs. *C. albicans*, or the fungal cell wall component β-glucan, enhanced monocytic proinflammatory cytokines production after a secondary unrelated stimulation [10]. Endogenous oxidized low-density lipoprotein (oxLDL) induced a long-lasting proinflammatory phenotype in monocytes, which has been associated with atherosclerosis and foam cell formation [11]. Also, myeloid cells of mice fed a western-type diet exhibited a long-lasting trained immunity phenotype [12]. Other inducers of trained immunity have been identified, such as extracellular labile heme that increased mouse survival in a polymicrobial sepsis model [13].

Innate immune memory is supported by epigenetic modifications affecting gene accessibility, and thus conferring long-term potential for tailored gene transcription, which promote a quick and heightened immune response [14]. Epigenetic memory has been reported not only in short-lived peripheric innate immune cells, but also in their long-lived progenitors. Inducers of innate immune memory skew bone marrow hematopoietic stem cells (HSC) differentiation towards myelopoiesis, with production of innate immune cells with increased effector functions. Indeed, HSCs of mice exposed to lipopolysaccharides exhibited conserved epigenetic marks for at least 12 weeks, which increased the expression of immune-related genes after a subsequent infection with *Pseudomonas aeruginosa* [15]. Similarly, BCG or β-glucan administration conferred protection of mice against tuberculosis and chemotherapy-induced myeloablation, respectively, via changes at the levels of HSCs [16, 17]. The establishment of long-term central innate immune memory was also shown in a human BCG vaccination model, wherein HSCs were transcriptionally distinct 90 days after BCG vaccination when compared to prior to vaccination [18]. The epigenetic changes of progenitor cells may underline the long-lasting protection of inducers of trained immunity. However, changes of progenitor compartments may also lead to a long-term maladaptive innate immune memory, with exacerbated inflammatory responses, such as in patients with atherosclerosis [19] or even after severe COVID-19 infection [20]. Also, an experimental model of periodontitis-induced maladaptive myelopoiesis, which rendered the mice more susceptible to arthritis, pointed to inappropriate induction of trained immunity in the bone marrow progenitors as an amplifier of inflammatory comorbidities [21]. In addition to HSC memory, epigenetic changes in mice have also been linked to the transmission of trained immunity traits through generations [22]. Specifically, the progeny of male mice previously infected with *C. albicans* were protected against heterologous infections. This protection derived from differences in DNA methylation are present in the sperm DNA of the infected parental mice [22].

Alterations to the epigenetic landscape are interwoven with metabolic changes, which together influence the establishment of innate immune memory. The integration of both extracellular metabolites from the microenvironment and intracellular metabolites with distinct transcriptional programs contribute to the establishment of innate immune memory. Metabolites act as signalling molecules, cofactors and substrates that modulate protein activity and ultimately contribute to cell function. In this review, we discuss the metabolic pathways that support the initiation and maintenance of innate immune memory, which are tightly connected with the molecular and epigenetic control of trained immunity. Here, we also suggest that metabolism is an attractive target for future therapeutic strategies, either to potentiate immune responses in immunosuppressed conditions or to decrease maladaptive activation of the innate immune system in hyperinflammatory diseases.

## Metabolic Modulation Supports Trained Immunity

**Fig. 2:**
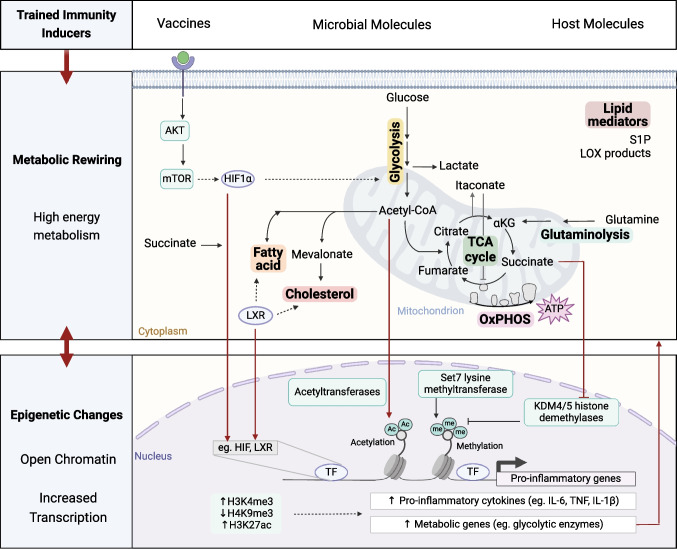
Metabolic pathways that support innate immune memory and their interaction with epigenetic regulators. The upregulation of multiple metabolic pathways supports the establishment of trained immunity in distinct cell types by providing energy, deferential building blocks and by modulating protein activity. Metabolites enriched upon trained immunity may activate transcription factors and modulate the activity of epigenetic enzymes. These promote an epigenetic signature that enables the increased transcription of pro-inflammatory and metabolic genes, which in turn amplify the existing metabolic shift and ultimately confer a sustained increase in effector functions. (S1P sphingosine-1-phosphate; LOX lipoxygenase; αKG α-ketoglutarate; TCA tricarboxylic acid; OxPHOS oxidative phosphorylation; AKT protein kinase B; mTOR mammalian target of rapamycin; HIF1α hypoxia inducible factor 1α; LXR liver X receptor; TF transcription factor; Ac acetyl; me methyl). Figure created with Biorender.com

Immune cells are finely attuned to extracellular cues and are able to quickly respond to microenvironmental changes. The rapid rewiring of metabolic pathways produces alterations in the bioenergetic profile of the cell and promotes the synthesis of signaling metabolites that ultimately affect cellular functions. Innate immune memory has been shown to be supported by different metabolic pathways, such as glycolysis, oxidative phosphorylation (OXPHOS), the tricarboxylic acid (TCA) cycle, amino acid, and lipid metabolism **(**Fig. 2**)**.

### Glycolysis and Oxidative Phosphorylation

**Fig. 3:**
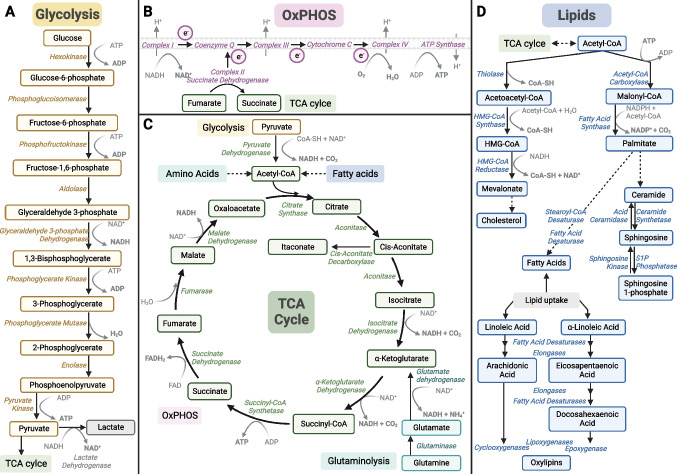
Schematic overview of the major metabolic pathways involved in innate immune memory, such as (**a**) glycolysis, (**b**) oxidative phosphorylation (OxPHOS), (**c**) tricarboxylic acid (TCA), glutaminolysis and (**d**) fatty acid, cholesterol, sphingolipid and oxylipin synthesis. Figure created with Biorender.com

Glycolysis involves the rapid degradation of glucose with production of pyruvate, energy and reducing power in the form of adenosine triphosphate (ATP) and nicotinamide adenine dinucleotide (NADH) respectively. Pyruvate can then fuel the TCA cycle or be oxidized into lactate (Fig. 3A). The upregulation of glycolysis, with increased glucose consumption and release of lactate, has been described in distinct cell types and in response to different inducers of innate immune memory. For example, glycolysis was upregulated in neutrophils isolated from BCG vaccinated individuals, revealing increased amounts of lactate secretion. Enhanced lactate production was correlated with enhanced fungicidal activity, possibly due to the associated increase in the release of reactive oxygen species (ROS) [23]. ROS were also increased in neutrophils of mice exposed to β-glucan [24]. BCG, β-glucan and oxLDL have also been shown to induce glycolysis in monocytes [25–27]. Indeed, the concomitant pharmacological inhibition of glucose uptake and exposure to the BCG or oxLDL led to the long-term decrease of responsiveness of human trained macrophages, while the inhibition of glycolysis of control cells did not affect their long-term cytokine production capacity [26, 27]. In addition, glycolysis may also be relevant in the establishment of central innate immune memory. Namely, hematopoietic stem cells of mice treated with β-glucan presented an increased glycolytic rate, and administration of β-glucan together with a glucose uptake inhibitor decreased the myeloid bias of hematopoietic stem cells characteristic of trained mice [17]. This increase in glycolysis was associated with changes in the epigenome, namely the increased deposition of the transcriptional permissive mark H3K4me3 at regions associated with the promoters of glycolytic enzymes [25–27]. The upregulation of glycolytic genes might be regulated by the protein kinase B (AKT) – mammalian target of rapamycin (mTOR)—hypoxia inducible factor (HIF)-1α pathway, ultimately contributing for the increased responsiveness characteristic of a trained cell.

Indeed, inhibition of mTOR with rapamycin or of HIF-1α with ascorbate decreased the pro-inflammatory cytokine production of β-glucan or BCG treated monocytes [25, 26]. Also, the myeloid specific deletion of *HIF1A* compromised the survival of β-glucan trained mice to a *S. aureus* infection [25]. In humans, oral metformin treatment decreased the ex vivo induction of BCG- and oxLDL-induced trained immunity in monocytes [26, 27]. Metformin is an antihyperglycemic drug known to inhibit mTOR through activation of AMP-activated protein kinase (AMPK). In addition, it also dampens oxidative phosphorylation (OXPHOS), by inhibition of complex I of the electron transport chain. It is important to highlight that mTOR and HIF-1α not only have a role in the activation of glycolysis but can also modulate other pathways. For example, chromatin immunoprecipitation followed by sequencing analysis of HIF-binding sites has identified approximately 500 sites across the genome [28]. At the metabolic level, HIF-1α has been shown to decrease the transcription of the TCA cycle enzyme succinate dehydrogenase (SDH) [29]. HIF-1α may also enhance the expression of glutamine transporters, thus increasing the intracellular concentration of glutamate [30]. Glutamate in turn may be converted to α-ketoglutarate, a metabolite of the TCA cycle. Of note, the consumption of glutamine to feed the TCA cycle, also known as glutaminolysis, has also been shown to play a role in innate immune memory, as discussed in the later sections of this review.

OXPHOS includes the oxidation of NADH, the creation of a proton gradient which is used by F0F1-ATP synthase to synthesize ATP, and the reduction of oxygen (Fig. 3B). Innate immune memory rely on a highly energetic metabolism, with the concomitantly increase of glycolysis and oxygen consumption, as observed for BCG, oxLDL and β-glucan [26] [27] [31]. Indeed, monocytes treated with the ATP synthase inhibitor oligomycin, followed by exposure to memory inducing stimuli such as β-glucan or oxLDL, presented a decreased long-term cytokine producing capacity when compared to trained macrophages that were not exposed to oligomycin [31, 32]. The potential role of OXPHOS in innate immune memory was also highlighted by suggestive associations between common single nucleotide polymorphisms (SNPs) in OXPHOS-related genes and the variability in the responsiveness of β-glucan- or oxLDL- trained monocytes [31, 32].

The increased mitochondrial metabolism of innate memory cells contributes to the increase in energy stores but also potentiates the production of mitochondrial ROS. Indeed, human monocytes exposed to oxLDL exhibited higher levels of mitochondrial ROS [33]. Similarly, neutrophils of zebrafish previously exposed to the bacteria *Shigella* respond to stimulation with higher levels of mitochondrial ROS, when compared to naïve neutrophils [34]. A sublethal dose of *Salmonella enterica* also generated neutrophils that respond to stimulation by producing increased amounts of mitochondrial ROS [35]. In this study, *S. enterica* infection rewired HSC towards the generation of neutrophils with enhanced bactericidal activity, which was phenocopied by overexpression of the transcription factor CCAAT/enhancer binding protein beta (C/EBPβ). C/EBPβ was also involved in LPS-induced memory of mice HSC, by targeting myeloid enhancers and potentially promoting myelopoiesis [15].

In addition to monocytes and neutrophils, lymphoid cell populations such as the natural killer (NK) cells have also shown memory characteristics. NK cells isolated from BCG-vaccinated individuals have also shown increased proinflammatory cytokine production following ex vivo heterologous stimulation [36]. Curiously, NK cells of human cytomegalovirus (HCMV) seropositive individuals exhibit heightened glycolysis, mitochondrial oxidative metabolism, and mitochondrial ROS, relative to NK cells of HCMV-seronegative individuals. This metabolic rewiring and increase in the capacity to produce IFN-γ was dependent on a chromatin modifying transcriptional regulator [37]. Thus, NK cell memory is potentially regulated by metabolic and epigenetic changes, similarly to monocytes [38].

Together, these findings suggest a relevant role for glycolysis, OXPHOS and also mitochondria metabolism and function in innate immune memory.

### Tricarboxylic Acid Cycle

The increase of oxidative phosphorylation and TCA cycle activity is characteristic of different trained immunity programs. The TCA cycle is a fundamental mitochondrial process that, through a series of oxidative reactions, reduces NADH and FADH_2_ coenzymes, contributing directly to the electron transport chain and ATP production (Fig. 3C). The TCA cycle also integrates several anabolic and catabolic pathways and produces metabolic intermediates [39]. Trained immunity is supported by the accumulation of TCA cycle intermediate metabolites. Upon induction and maintenance of trained immunity, two main carbon sources are responsible for the supplementation of TCA cycle metabolites: (i) pyruvate-derived acetyl-CoA and (ii) glutamine-derived α-ketoglutarate.

The former derives from the upregulation of glycolysis, which produces increased levels of pyruvate. Once imported into the mitochondria, pyruvate is oxidized into acetyl-CoA. β-oxidation of fatty acids also feeds the TCA cycle by producing acetyl-CoA and NADH and FADH_2_. Acetyl-CoA not only fuels the TCA cycle but also acts as a source of acetyl groups for histone acetyltransferases. Indeed, the increase in the intracellular concentration of acetyl-CoA promotes histone 3 lysine 27 acetylation (H3K27ac) and leads to the upregulation of glycolysis-related genes, such as hexokinase 2, phosphofructokinase and lactate dehydrogenase [40]. Particularly, a short exposure to exogenous acetyl-CoA increased the capacity of human monocytes to produce IL-6 upon TLR2 stimulation [41]. Acetyl-CoA can be metabolised and condensed with oxaloacetate to form the TCA cycle intermediate citrate. Citrate levels in stimulated macrophages are central for the induction of proinflammatory factors such as ROS, nitric oxide (NO), and prostaglandins [42]. In the cytosol, citrate can also be cleaved to regenerate Acetyl-CoA and used as a precursor of lipid biosynthesis.

In addition to glycolysis, glutaminolysis fuels the TCA cycle and is necessary for the establishment of trained immunity [43]. Glutamine is converted to glutamate, which in turn is synthesised into alpha-ketoglutarate (α-KG) (Fig. 3C). α-KG is a cofactor necessary for the activity of α-KG dependent dioxygenases (α-KGDD). However, trained monocytes are also characterized by the accumulation of the TCA cycle metabolites succinate and fumarate. These metabolites compete with α-KG and inhibit the activity of multiple α-KGDD [44]. Among the α-KGDD family are the prolyl hydroxylase domain (PHD) enzymes, which hydroxylate the transcription factor HIF-1α, promoting its constitutive degradation. Stabilization of HIF-1α is crucial for the induction of trained immunity, promoting the induction of IL-1β transcription, glycolysis, and expression of histone demethylases [25, 45, 46].

The α-KGDD family also includes histone and DNA demethylases. Particularly, fumarate accumulation in human monocytes was shown to increase the production of pro-inflammatory cytokines after stimulation, while also increasing the deposition of H3K4me3 at regions associated with the promoters of the pro-inflammatory genes *TNF* and *IL6*. The enrichment of this transcriptional-permissive H3K4me3 mark was associated with a fumarate-induced decrease in KDM5 activity, a histone H3K4 demethylase [43]. Other α-KGDD have also been implicated in the establishment of innate immune memory. The pharmacological inhibition of the H3K9 histone demethylase KDM4 decreased glycolysis and the capacity of β-glucan trained human monocytes to secrete IL-6 and TNF upon TLR4 stimulation. Accordingly, inhibition of KDM4 increased the deposition of the repressor mark H3K9me3 at regions associated with promoters of *IL6* and *TNF* of β-glucan trained cells [47]. KDM4 expression was also increased in stimulated myeloid progenitor cells of mice trained with a western-type diet [12]. This interplay between metabolic intermediators and the activity of epigenetic enzymes illustrates the cross-regulation of metabolism and epigenetics that underlines innate immune memory [14, 48].

The upregulation of the TCA cycle promotes an accumulation of succinate in trained monocytes [49]. Succinate can be oxidised to fumarate by succinate dehydrogenase (SDH), also known as complex II of the electron transport chain. SDH is a bridge enzyme between the TCA cycle and the OXPHOS, since the oxidation of succinate into fumarate promotes the transference of an electron along the electron transport chain. Genetic human studies have revealed the presence of individual SNPs in SDH genes, which were associated with changes in the capacity of pro-inflammatory cytokine production [49]. Furthermore, the transcription of *SDHB* and *SDHD* were increased in human macrophages trained with β-glucan [31]. The increase in SDH transcription was accompanied by the enrichment of TCA cycle metabolites, which were ablated by the pharmacological inhibition of the lysine methyltransferase Set7. Similarly, bone marrow cells of mice exposed to β-glucan exhibited a long-term increase in *SDHB* that was not observed in β-glucan-trained *SET7* deficient animals. The transcription of *SDHB* appears to be regulated by the acquisition of H3K4me1 at *SDHB* enhancer regions which is deposited by the activity of Set7 induced by β-glucan [31]. These data highlight that the enzymes that participate in the TCA cycle are also regulated at the epigenetic level, particularly through the action of Set7.

SDH activity is not only regulated at the transcriptional but also at the protein level, namely by Itaconate, a competitive inhibitor of SDH [50]. Itaconate is produced by aconitate decarboxylase 1 (ACOD1) from cis-aconitate, an intermediate of the TCA cycle. It is synthesised in pro-inflammatory macrophages [51] and triggers anti-inflammatory and antioxidant responses [52–54]. Itaconate not only inhibits SDH but may also affects other metabolic pathways by reacting with thiol groups [55]. Itaconate and its derivatives have been reported to alkylate the ROS scavenger molecule glutathione [53], and different glycolytic enzymes [52, 55, 56]. In line with the anti-inflammatory activity of itaconate, β-glucan restored the cytokine production capacity of LPS-tolerized monocytes, possibly by inhibiting itaconate production and re-establishing SDH function [49]. Also, supplementation of monocytes with the derivative dimethyl itaconate led to the inhibition of β-glucan-induced trained immunity [49]. In contrast, monocytes exposed to dimethyl itaconate in the absence of other stimuli exhibited features of trained immunity, with metabolic, transcriptional, epigenetic and pro-inflammatory changes similar to those induced by the prototypical inducer of trained immunity β-glucan [57]. DMI administration also increased mice survival to *S. aureus* infection. Dimethyl itaconate-induced trained immunity possibly involved glutathione metabolism. Glutathione was shown to also be involved in vitro β-glucan-induced trained immunity [58] and its pharmacological inhibition reduces the cytokine production of both DMI- and β-glucan-trained macrophages [57, 58]. Itaconate may also modulate the epigenetic landscape of innate immune cells. Of note, itaconate was shown to inhibit TET DNA dioxygenases upon LPS stimulation, and may also suppress the activity of other α-KGDD [59]. However, its role in the epigenetic control of trained immunity has not been addressed. Overall, itaconate and its derivatives have a dual anti- and pro-inflammatory role in trained immunity that is context dependent.

### Amino acids

Amino acids have a central role in cellular metabolism and innate immunity activation. Amino acids are not only the building blocks of proteins but also act as precursors of metabolites involved in the trained immunity phenotype. As discussed above, glutaminolysis is essential for trained immunity induction since it replenishes the TCA cycle via α-ketoglutarate. Indeed, pharmacological inhibition of glutaminase blunted the stimulated production of IL-6 and TNF by β-glucan-trained human monocytes. In line with this, glutaminolysis inhibition also decreased the deposition of H3K4me3 at regions associated with the promoters of the *IL6* and *TNF* genes [43]. In addition to glutamine, human monocytes exposed to β-glucan also exhibited increased consumption of aspartate and methionine [43], however, the role of these amino acids in the establishment of innate immune memory remains to be explored further. Methionine, in particular, is the precursor of S-adenosylmethionine (SAM), a methyl donor critical for DNA and histone methylation. LPS-stimulated macrophages upregulate the methionine cycle to fuel the production of SAM which in turn contributes to histone methylation that promotes IL-1β production [60]. Different amino acids have been shown to influence the activation of innate immune cells, however their contribution to the establishment of memory is underexplored. More studies are warranted to obtain a clearer understanding of their specific role in both immunometabolism rewiring and epigenetic modifications driving innate immune memory.

### Lipid metabolism

Lipid metabolism has also been implicated in different programs of innate immune memory. Cells acquire fatty acids through both de novo lipogenesis and by uptake from the environment (Fig. 3D). Dietary fatty acids can act as inflammatory stimuli and their involvement in trained immunity has been reported [61]. A diet rich in saturated fatty acids altered the relative populations of hematopoietic stem cells in mice and increased the responsiveness of bone marrow and circulating monocytes to ex vivo TLR4 stimulation. Particularly, the saturated fatty acid palmitate contributed to the increased response of macrophages, and palmitate exposure was sufficient to enhance the clearance of *C. albicans* from infected mice that lack mature T and B cells [61]. Similarly, a genetic mouse model of atherosclerosis fed a high-fat diet, which induces transient hypercholesterolemia, exhibited a shift of the hematopoietic compartment towards an enrichment of granulocyte-monocyte progenitors [12]. This myeloid bias was sustained, even after the mice returned to a standard chow diet and the cholesterol levels normalized. These results point to the capacity of dietary compounds to modulate innate immune responses. Of note, a high-salt diet was also recently shown to induce a persistent immune memory imprinted in the hematopoietic compartment, with a transcriptional increase of genes involved in glycolysis and fatty acid metabolism [62].

In addition to dietary enrichment of cholesterol, the intracellular upregulation of cholesterol pathways has also been implicated in trained immunity. Namely, various species of cholesterol esters were enriched in hematopoietic stem cells isolated from β-glucan-trained mice. In this model, the pharmacological inhibition of the cholesterol pathway decreased the hematopoietic myeloid bias induced by β-glucan [17]. Mevalonate, synthetized from acetyl-CoA, may be used for the production of cholesterol and it is thought to be the bioactive metabolite of this pathway in the induction of innate immune memory. Accordingly, the inhibition of mevalonate production decreased lactate secretion, H3K4me3 deposition at regions associated with the promoter of *TNF* and ultimately the production of TNF, which were enhanced in BCG- or oxLDL- trained monocytes [63]. In line with this, a constitutive trained immunity phenotype was observed in hyper immunoglobulin D syndrome patients, who present with mevalonate accumulation [63]. Thus, mevalonate has been shown to induce maladaptive trained immunity, that leads to the sterile inflammatory attacks observed in these patients, while also contributing to the protection conferred by different stimuli such as β-glucan, BCG and oxLDL [17, 63].

A master regulator of fatty acid metabolism, the nuclear liver X receptor (LXR), was shown to also control the induction of trained immunity. The pharmacological inhibition of this transcription factor decreased the secretion of IL-6 and TNF by stimulated macrophages previously exposed to oxLDL or BCG [41, 64]. LXR inhibition also dampened the metabolic and epigenetic rewiring induced by oxLDL-induced trained immunity, as observed with the decrease in lactate secretion and the reduced H3K4me3 deposition at regions associated with the promoters of *IL6* and *TNF* [64]. Interestingly, the activation of LXR was sufficient to phenocopy the increased responsiveness, metabolic and epigenetic changes of a trained immune cell. Particularly, the increased capacity for IL-6 and TNF secretion induced by LXR activation was dependent on the mevalonate pathway [41]. Thus, highlighting the different contexts of innate immune memory activation in which mevalonate participates.

Trained immunity has also been associated with other lipid metabolic pathways. A lipidomic analysis performed of hematopoietic progenitors isolated from β-glucan-trained mice revealed an enrichment for shorter and more saturated fatty acids, and a decrease in lipids containing arachidonic acid [17]. In contrast, a recent study reported that long-chain polyunsaturated fatty acids support BCG-induced increased responsiveness in human monocytes [65]. In this study, arachidonic acid levels were not modulated by β-glucan or BCG, instead, a tailored production of lipid mediators was observed. Particularly, lipid mediators produced by lipoxygenases (LOX) were enriched in BCG exposed monocytes, and the inhibition of 5- or 12-LOX activity dampened the effect of BCG on cytokine production. Interestingly, 12-LOX derived lipid mediators were increased in monocytes isolated from individuals that had been vaccinated one month prior with BCG [65]. Lipid mediators are produced through the enzymatic oxidation of polyunsaturated fatty acids and a wealth of studies have lately underscored that they are involved in different phases of host defense, from inflammation and induction of innate immunity, to modulation and resolving processes [66]. From that perspective it is not surprising that they are involved in the induction and modulation of trained immunity, and they could potentially represent therapeutic targets in diseases in which trained immunity is involved. In addition to LOX-derived lipid mediators, another lipid mediator has also been implicated in the induction of trained immunity. Sphingosine-1-phosphate abundance was increased in whole particle β-glucan exposed murine macrophages, and inhibition of the production of sphingosine-1-phosphate attenuated TNF secretion [67]. The authors suggest that sphingosine-1-phosphate may promote mitochondrial fission, which in turn was essential for the increased responsiveness and oxygen consumption triggered by whole particle β-glucan [67]. In accordance, a recent study also reported that pharmacological modulation of sphingolipid metabolism affected *C. albicans*-induced trained immunity in human monocytes [68]. Particularly, inhibition of the enzyme acid ceramidase, which converts ceramide to sphingosine, dampened the boosted cytokine production, glycolytic rate and OxPHOS potentiated by *C. albicans.*

## Local Innate Immune Memory: the Case of the Lung

Innate immune memory has mostly been explored in a systemic context, in which peripheral innate memory cells are continuously replenished by HSC that retain epigenetic memory [16–19, 21]. However, some studies suggest that innate immune memory is also induced and maintained in self-contained tissues [69, 70]. Particularly, respiratory infections have been a focus of study, possibly due to the preferential heterologous protection provided by BCG vaccination in humans [4, 7]. Particularly, alveolar macrophages (AMs), the resident macrophages of the airways, have been cells of interest since they act as the primary immune sentinels of the respiratory tract. However, it is important to note that resident AM originate from the yolk sac during embryonic haematopoiesis, and not from circulating monocytes [71]. Thus, there are subsets of lung tissue-resident macrophages that have different ontologies and possibly distinct immunological functions [72]. Consequently, it is important to employ strategies that allow for accurate cellular identification that consider the similarities between monocytes recruited to the lung and the resident AM population. Not only ontology but also environment may affect cell function. It is relevant to highlight that glucose concentrations in the airways are lower than in circulation [73], which might influence the metabolic pathways employed by the resident cells and not fully recapitulate the metabolic rewiring that takes place in other tissues or culture media conditions.

In mouse models, acute respiratory viral infections have been shown to induce memory features in alveolar macrophages (AM), protecting mice from subsequent bacterial infections [74] and promoting long-term antitumor immunity [75]. In particular, trained AM demonstrated a markedly increased glycolytic metabolism, which was ablated by T cells depletion or IFNγ deficiency. The authors reported that IFN-γ from effector CD8 + T cells was necessary for AM memory formation [74]. Similarly, IFN-γ has also been shown to be crucial for central trained immunity, since HSC of IFN-γ-receptor deficient mice failed to expand upon BCG vaccination [16]. AM with long-term memory induced by viral infection also exhibited increased oxygen consumption rates, which were fuelled by fatty acids and glucose oxidation [75]. Fatty acid oxidation has also been shown to participate in AM memory formation upon mouse exposure to ambient amounts of lipopolysaccharides [76]. Another murine study reported that LPS inhalation induced trained immunity in AM, which exhibited increased efferocytosis efficiency and transcriptional upregulation of lipid and arginine metabolism, ultimately enhancing inflammation resolution [77].

In addition to the local triggering of innate memory, the increase in lung immunity has also been reported upon systemic induction of trained immunity. BCG subcutaneous immunization of mice led to increased glycolysis and oxidative phosphorylation of AM [78]. However, it also altered intestinal microbiota, and promoted the levels of intestinal short-chain fatty acids (SCFA) carnitine and butyrate. The authors reported that this enrichment in the intestine was similarly found in the serum and lungs of BCG vaccinated animals. Via microbiota transplantation models or by introducing SCFA in the drinking water of naïve mice, the authors suggest that there is a causal relationship between BCG-altered microbiota and AM memory. Curiously, the gut microbiome has also been implicated in innate immune memory features in humans. In a human BCG vaccination cohort, the abundance of *Roseburia* in stool samples negatively correlated with ex vivo circulating innate immune responsiveness, possibly due to the modulation of plasma phenylalanine metabolism [79]. Particularly in human AM isolated from sputum, BCG vaccination decreased the expression of surface activation markers, but the functional consequences remain to be investigated [80].

## Implications for Disease and Future Therapies

**Fig. 4:**
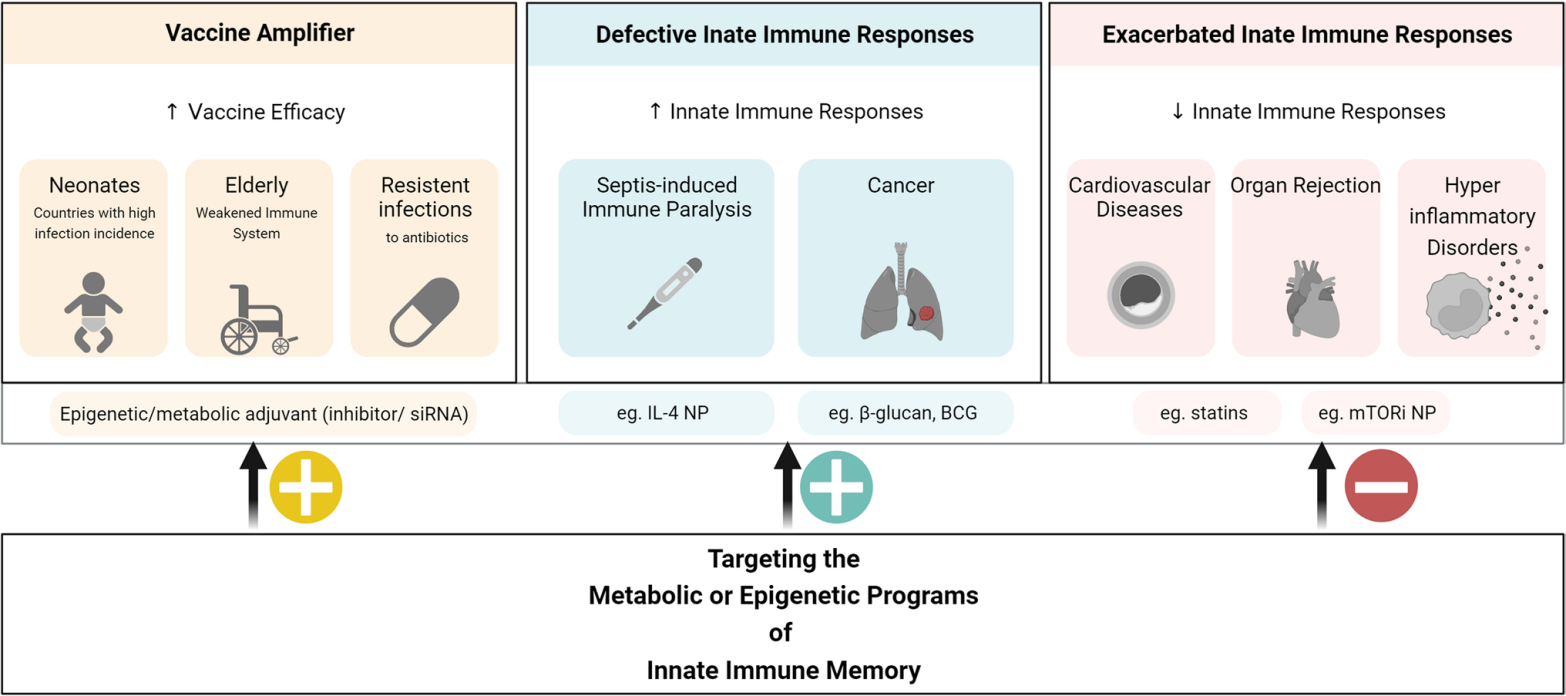
Clinical scenarios that might benefit from the modulation of innate immune memory and possible therapeutic approaches. The therapeutic use of innate immune memory may encompass the improvement of vaccines and the regulation of compromised or hyperactive immune responses. The design of vaccine amplifiers that trigger innate immune memory might not only enhance the pathogen-specific responses induced by the classical vaccine formulations, but also provide cross-protection to unrelated infections. This increased non-specific protection would be of particular interest in countries of high infection burden and to counter the rise in antibiotic resistant pathogens. Individuals with a weakened immune system could also benefit from a boosted innate immunity, such as in the case of sepsis or cancer. However, the dampening of innate immune memory could also be a strategy in conditions of exacerbated immune responses, as for example in cardiovascular diseases and graft versus host disease. In order to target innate immune memory programs, the use of prototypical inducers such as BCG or β-glucan has been suggested. Also, the use of metabolic or epigenetic modulators has been hypostatised. The targeted delivery of mRNA, siRNA, inhibitors, or cytokines could be performed by nanoparticles (NP) carries administered locally or systemically. Figure created with Biorender.com

The growing field of trained immunity, highlighted by metabolic changes in myeloid and lymphoid cells, offers new treatment options for many diseases where imbalanced immune responses play a key role. The close relationship between cellular metabolism and trained immunity not only clarifies the mechanics of immune responses, but also suggests a new approach for therapeutic interventions. Drug-based modulation, similar to vaccination strategies used in the adaptive immune system, may have potential in using the memory-like properties of the innate immune system for disease prevention and treatment. This immunometabolic connection outlines a hopeful path for therapeutic targeting, where metabolic pathways and their interactions with immune responses can be adjusted to lessen or prevent disease conditions (Fig. 4).

### Heterologous Effects of BCG Vaccination

As outlined in previous sections, the BCG vaccine induces non-specific protection against heterologous infections through the induction of trained immunity. A study involving 325 individuals demonstrated that BCG vaccination influences circulating metabolite concentrations which, in turn, impact trained immunity responses such as increased IL-1β and TNF production upon stimulation with the gram-positive bacteria *Staphylococcus aureus*. The metabolic foundation of trained immunity encompasses more than just glycolysis and the tricarboxylic acid cycle, extending to lipid and amino acid metabolism, thus illustrating a complex metabolic network driving immune responses. In this regard, taurine metabolism was also identified as a new metabolic pathway associated with BCG-induced trained immunity [81]. Notably, alterations in cholesterol metabolism, a key player in atherosclerosis, have also been connected to the modulation of trained immunity, suggesting a potential therapeutic target. The application of statins, known for regulating cholesterol levels, might impact trained immunity and contribute to atherosclerosis management. However, an interventional study in patients with familial hypercholesterolemia showed that whereas monocytes from these patients present a trained immunity phenotype, treatment with statins does not revert this pro-inflammatory phenotype [82].

### Vaccine Adjuvants as Innate Immune Amplifiers

The advent of pharmacological amplification posits a transformative approach to bolster the effectiveness of vaccines, focusing on the fine-tuning of metabolic and epigenetic mechanisms within immune cells. This paradigm leverages the modulation of metabolic pathways, to augment both specific and nonspecific immune responses post-vaccination. For instance, the modulation of glycolysis through compounds like 2-deoxyglucose, or TCA cycle and OXPHOS through substances like dichloroacetate [83], showcases a prospect of enhancing immune responses.

In the global fight against antimicrobial resistance (AMR), vaccines stand as a formidable first line of defence, with the potential to drastically reduce the utilization and thus resistance to antibiotics. This could be achieved through the addition of molecules that are able to modulate the most important metabolic pathways and potentiate trained immunity responses. This idea extends to providing a robust immune response against emerging infections, even when specific vaccines are yet under development, thus acting as a bridge to curb morbidity and mortality during the interim. The envisioned new generation of vaccines, accentuated with metabolic amplifiers, aims at tackling both communicable and non-communicable diseases. The "amplifier" component in vaccines, as proposed, would be able to enhance the memory and effector functions of immune cells, broadening the scope of vaccine efficacy beyond specific antigenic targets to a wider array of pathogens, including those resistant to current antimicrobial treatments. The implications of this novel vaccination strategy resonate profoundly in populations with compromised immune responses such as the elderly and immunocompromised individuals. The underlying principle of augmenting innate immune responses through metabolic modulation could provide an adjuvant strategy to the diminished vaccine efficacy in these groups. The utilization of live vaccines like BCG has shown promise in enhancing immune responses in the elderly, indicating the potential of trained immunity in this demographic [7]. As the world grapples with multiple infectious diseases, alongside an increasing prevalence of AMR, the exploration of metabolic modulators as vaccine amplifiers opens a promising avenue. The envisioned advancements in vaccine technology underscore a concerted effort towards unlocking the full potential of vaccines, aligning with a broader strategy to combat infectious diseases through metabolic tuning, thereby opening new horizons in the realm of immunotherapy and disease control.

### Potential of Nanomedicines

One significant technological development is the control of trained immunity through nanomedicine, which appears promising particularly in diseases like cancer and inflammation-related syndromes. The control achieved through epigenetic and metabolic reprogramming of hematopoietic stem and progenitor cells underlines the potential of targeting metabolic pathways to adjust trained immunity for cancer treatment. The chance to deliver metabolic modifiers or immune-modifying agents accurately to specific cell types or tissues is promising for precisely adjusting trained immunity based on the situation. Nanoparticles that can deliver small molecule inhibitors, siRNA, or other agents could offer a way to adjust the metabolic and epigenetic foundations of trained immunity with high accuracy and low systemic toxicity.

For instance, the phenomenon of sepsis and the resulting immune paralysis significantly highlight the intricate role of metabolic regulation in trained immunity. In scenarios of sepsis, a state of immune paralysis often ensues, marked by a compensatory anti-inflammatory response syndrome which augments the risk of secondary infections and death [84]. The process of metabolic reprogramming is pivotal in directing immune responses during sepsis, and in turn, modulating the state of immune paralysis. A recent study used nanomedicine to target interleukin-4 (IL-4) to myeloid cells to alleviate sepsis-induced immunoparalysis, utilizing the principles of trained immunity. In cultured primary human monocytes, IL-4 inhibits acute inflammation, while simultaneously inducing a long-lasting innate immune memory. To harness this dual characteristic of IL-4 in vivo, a fusion protein of apolipoprotein A1 (apoA1) and IL-4 was developed, integrating the cytokine into a lipid nanoparticle. When injected intravenously, the apoA1-IL-4-embedding nanoparticle targets myeloid-cell-rich haematopoietic organs, particularly the spleen and bone marrow. The study further demonstrated that IL-4 nanotherapy resolved immunoparalysis in mice with lipopolysaccharide-induced hyperinflammation, as well as in ex vivo human sepsis models and in experimental endotoxemia. These findings advocate for the translational development of nanoparticle formulations of apoA1-IL-4 for the treatment of patients with sepsis who are at risk of immunoparalysis-induced complications [85].

The field of cancer provides a crucial setting to examine the complicated interaction between metabolic processes and trained immunity. The cancer-induced changes in the tumor microenvironment (TME) and systemic metabolism create a detailed scenario where trained immunity could act as both a foe and friend. A deep understanding of these dynamics is crucial for creating innovative therapeutic strategies. Metabolic reprogramming is a common feature in both cancer cells and immune cells. Within the TME, trained immune cells show altered metabolic profiles that can either encourage or halt tumor progression. Triggering trained immunity in pro-tumorigenic macrophages is also a potential strategy to manage tumor metastasis. In this regard, in vivo treatment with whole beta-glucan particle led to a trained immunity behaviour in lung interstitial macrophages, resulting in the blocking of tumor metastasis and extending survival in multiple metastatic mouse models. As discussed previously, this trained immunity was driven by the metabolite sphingosine-1-phosphate, highlighting the importance of metabolic intermediates in adjusting trained immunity and possibly using it for cancer treatment [67]. Furthermore, the immunological behaviour of trained immunity in cancer might change based on the level of metabolic reprogramming brought on by the extent of training, suggesting a complicated interaction between metabolic signals and trained immunity activation. Considering the anti-cancer findings, the metabolic reprogramming observed in alveolar macrophages (AMs) post-influenza infection, as depicted by elevated glycolysis, PI3K–Akt, HIF-1, and mTOR signalling, underscores the therapeutic promise of leveraging trained immunity against cancer. The enhanced oxygen consumption and glycolytic rate reflect a prolonged metabolic rewiring instrumental in fortifying antitumor responses [75]. These findings accentuate the potential of trained immunity, driven by metabolic adaptations, as a viable approach to bolster antitumor defences, thereby broadening the spectrum of cancer therapeutic strategies.

### Steps from bench to bedside

Given these findings, a deeper investigation into how metabolism regulates trained immunity in different diseases is necessary to develop new treatment strategies. The complex metabolic pathways linked with trained immunity suggest many potential targets for treatment. For example, targeting metabolic enzymes like glycolytic enzymes or key parts of lipid metabolism could have a modifying effect on trained immunity, and in turn, change the progression of a disease. Regulating trained immunity through metabolic pathways could also work alongside existing treatments to boost effectiveness and improve results in a wide range of diseases, including infectious diseases, autoimmune disorders, and cancers. New techniques like metabolomics and single-cell sequencing provide exceptional insights into the metabolic basis of trained immunity. These techniques allow for a detailed examination of the metabolic changes that come with trained immunity and possible implications in disease. For instance, identifying metabolic markers that characterize trained immune cells can reveal new targets for treatments. A deeper understanding of how metabolic pathways change in response to various immune challenges will help improve treatment strategies to adjust trained immunity.

Moreover, developing these ideas into clinical practice requires a strong regulatory framework to ensure the safety and effectiveness of treatments targeting trained immunity. The variation in metabolic reprogramming across different people and disease conditions is challenging and calls for a personalized approach to treatment. Discovery and validation of biomarkers will be crucial for categorizing patients and monitoring treatment responses. It is also important to consider the possible unintended effects and the long-term implications of adjusting trained immunity. A detailed understanding of the balance between immunological and metabolic factors is vital to avoid unexpected outcomes and to fully use the potential of trained immunity approaches.

In conclusion, the growing understanding of the close relationship between metabolism and trained immunity highlights new treatment possibilities. The ability to regulate trained immunity through metabolic interventions could lead to a new wave of treatments for a wide range of diseases. Ongoing research and collaboration across different disciplines will be crucial to uncover the full range of therapeutic possibilities offered by adjusting the metabolic aspects of trained immunity, thus marking a new phase of understanding and managing diseases.

## Data Availability

Not applicable for a review.
